# Combining Rubber Filings with Fresh Rubber

**Published:** 1894-04

**Authors:** W. S. Simonton

**Affiliations:** West Virginia


### COMBINING RUBBER FILINGS WITH FRESH RUBBER.

BY DR. W. S. SIMONTON, WEST VIRGINIA.

To many it may be difficult to imagine that filings and scrapings from finishing plates can be of any value, yet they can be made very useful. After the rough and dirty portions of the plates are scraped or filed off, save the balance, and in packing a thick heavy plate, first dip the rubber in warm water to soften, then roll it in the filings, and pack as usual; vulcanize carefully, and I will insure a good plate, well vulcanized, strong and tough and not porous. Rubber treated thus before packing vulcanizes in less time.

In heavy plates it is well, of course, to vulcanize carefully; but with ordinary care the plate will not become porous. I use this method in all plates, even where required to be made thin, as it vulcanizes five or ten minutes quicker, and I think produces better results. Formerly, in some very thick portions of plates, especially those that require much building upon the sides, it seemed almost impossible to prevent the thick heavy portions from becoming porous; all brands of rubber are alike in this respect, but since using this method I have not had one bad result or one porous plate. Porous plates have always been a source of trouble and much extra work. But this method of rolling each piece of rubber in the filings from finishing plates, I think, will be found to always prevent any trouble from this source. Try it, and if you have no filings saved and have a heavy plate to make, from an old plate make sufficient to pack the one on hand.

